# Management of Fire Arm Injury chest, an experience of 10,200 patients

**DOI:** 10.1186/1749-8090-10-S1-A15

**Published:** 2015-12-16

**Authors:** Amer Bilal

**Affiliations:** 1 Dept of Cardiothoracic Surgery, Lady Reading Hospital, Peshawar, Pakistan

## Background/Introduction

Firearm injury chest are among the most common and leading cause of trauma along with roadside accidents in tertiary care hospitals around the world. The situation in the developing world, including Pakistan, is even worse where terrorism, poverty, social inequality, unemployment and access to the illegal weapons are obvious.

## Aims/Objectives

To determine the surgical management of fire arm injury chest.

## Method

An observational descriptive study was conducted in the department of Thoracic Surgery, Postgraduate Medical Institute, Lady Reading Hospital Peshawar from June 2002 to Dec 2014. The record of all trauma patients that had fire arm injuries undergoing surgical intervention over a period of twelve years was reviewed.

## Results

The study included 10,200 patients; all were having firearm injuries leading to hemopneumothorax. Male to female ratio was 2:1. All patients were initially managed with tube thoracostomy. 400/10200 (3.92%) patients underwent emergency Thoracotomy, Rest of the patients i.e. 9801/10200 (96.07%) were hemodynamically stable and treated with low pressure suction and incentive spirometry. In 550/10200 (5.39%) patients which developed clotted hemothorax were evacuated successfully through thoracotomy. The mean time interval between injury and thoracotomy was 14.5 days (range between 11- 124 days). The mean volume of clotted hemothorax evacuated was 650 ml.The mean post-operative hospital stay was 5 days.

## Discussion/Conclusion

Majority of fire arm injuries were successfully managed by chest intubations, observation and supported treatment. 400 patients required emergency Thoracotomy, 550 patients went into develop clotted Hemothorax requiring evacuation

